# Application of a Dermal Regeneration Matrix for the Surgical Treatment of an Oromaxillary Defect in Medication-Related Osteonecrosis of the Jaw

**DOI:** 10.7759/cureus.35833

**Published:** 2023-03-06

**Authors:** Rui Seixas, Natacha Ribeiro, Ana Filipa Augusto, Carlos Matos, Manuel Tolentino

**Affiliations:** 1 Department of Stomatology, Hospital de São Bernardo - Centro Hospitalar de Setúbal, EPE, Setúbal, PRT; 2 Instituto Superior de Educação e Ciências, Instituto Superior de Educação e Ciências Lisboa, Lisboa, PRT; 3 Department of Maxillofacial Surgery, Centro Hospitalar e Universitário de Coimbra, EPE, Coimbra, PRT

**Keywords:** oromaxillary defect, mronj, jaw osteonecrosis, dermal regeneration matrix, bisphosphonates

## Abstract

Surgical options for managing patients with medication-related osteonecrosis of the jaw are multiple and may range from superficial debridement to vascularized osteocutaneous free flaps. However, some protocols may not be an option in specific cases, and alternative techniques for patients who are not likely candidates for more invasive procedures may represent a suitable solution to treat these patients. Here, we report the case of a 69-year-old man who presented to the Stomatology Department with dysphagia, exposed bone, and mild mandibular pain for the past three months. The patient was diagnosed with stage III medication-related osteonecrosis of the jaw. Surgical debridement with sequestrum removal was performed and the remaining intraoral defect, which was unsuitable for primary closure, was addressed with a dermal regeneration matrix. This system allowed full coverage of the surgical wound. At 10 weeks of follow-up, the intraoral defect had healed completely with no need for active intervention. Dermal regeneration matrixes may represent a surgical approach to cover oromaxillary defects in select patients.

## Introduction

Medication-related osteonecrosis of the jaw (MRONJ) is a critical side effect in the oromaxillary region, frequently presenting in oncological patients on bisphosphonates or antiangiogenic drugs over a long period. It is characterized by the exposure of necrotic gnathic bone or probing through a fistula that has persisted for at least eight weeks [1].

Treating MRONJ remains challenging due to the impaired healing and reduced angiogenesis of the affected bone [2]. Standard MRONJ treatments may require extensive surgical reconstruction with a free bone graft. If a more conservative approach is needed, long-term antibiotics with additional therapies may be applied [3]. However, the reduced ability for bone healing or the application of procedures that increase morbidity may lead patients to limited therapeutical options.

Research for additional therapeutic strategies includes the use of dermal matrixes such as Integra® Dermal Regeneration Template (IDRT, Integra Life Sciences, USA), which is a dual-layer skin regeneration matrix system to cover intraoral defects. IDRT is reported in the literature with multiple applications [4], and the available results show good healing for reconstructive surgery [5]. Nevertheless, only one report has demonstrated the use of the matrix in the management of MRONJ and its clinical evolution [6]. Here, we present the case of a 69-year-old man with an oromaxillary defect due to MRONJ who was treated with a dermal matrix.

## Case presentation

A 69-year-old man was referred to the Stomatology Department with complaints of dysphagia, exposed bone, and mild mandibular pain for the past three months. His medical history included an infrarenal aorta aneurysm, a prostate carcinoma with bone metastasis treated with androgen deprivation therapy, intravenous zoledronic acid 4 mg every four weeks for two years, and a lung adenocarcinoma treated with a lobectomy with mediastinal lymphadenectomy and cisplatin. The usual medication regimen included bicalutamide, naftidrofuryl, acetylsalicylic acid, amiodarone, furosemide, omeprazole, lorazepam, and beclomethasone.

**Figure 1:**
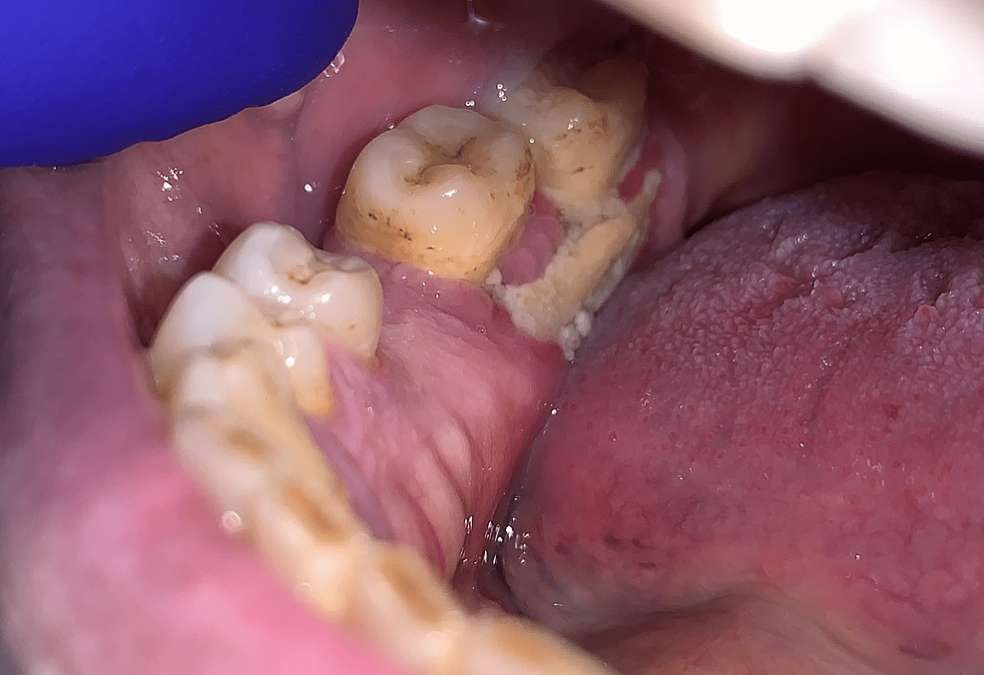
Preoperative clinical evaluation revealing exposed bone at the lower right molar region.

On clinical inspection, two exposed bone lesions were observed first, a draining fistula in the maxilla in close relation to 17, and, second, a bony sequestrum at the lingual area of the right molar region (Figure 1).

**Figure 2:**
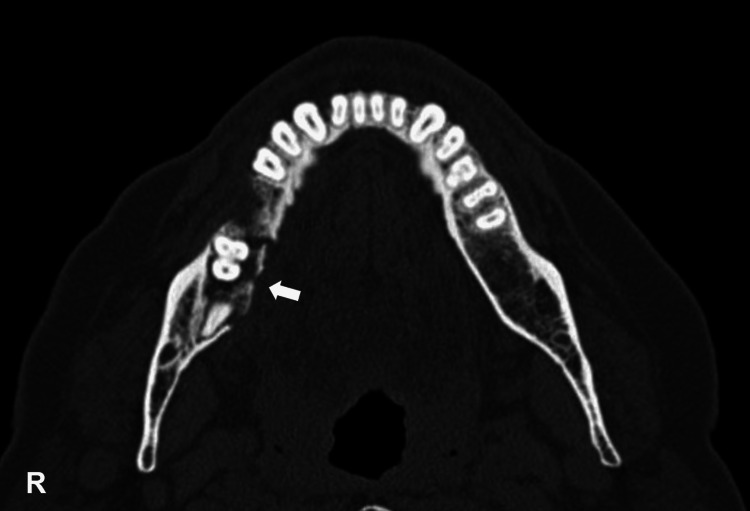
On axial CT in the bone window setting, a lytic lesion with sequestrum formation (arrow) can be seen in the posterior region of the right body of the mandible. R = right

This observation allowed the classification as a stage III MRONJ. CT confirmed the presence of a bone sequestrum on the lingual cortical plate in relation to teeth 47 and 48 with 4 cm of the major axis (Figure 2).

**Figure 3:**
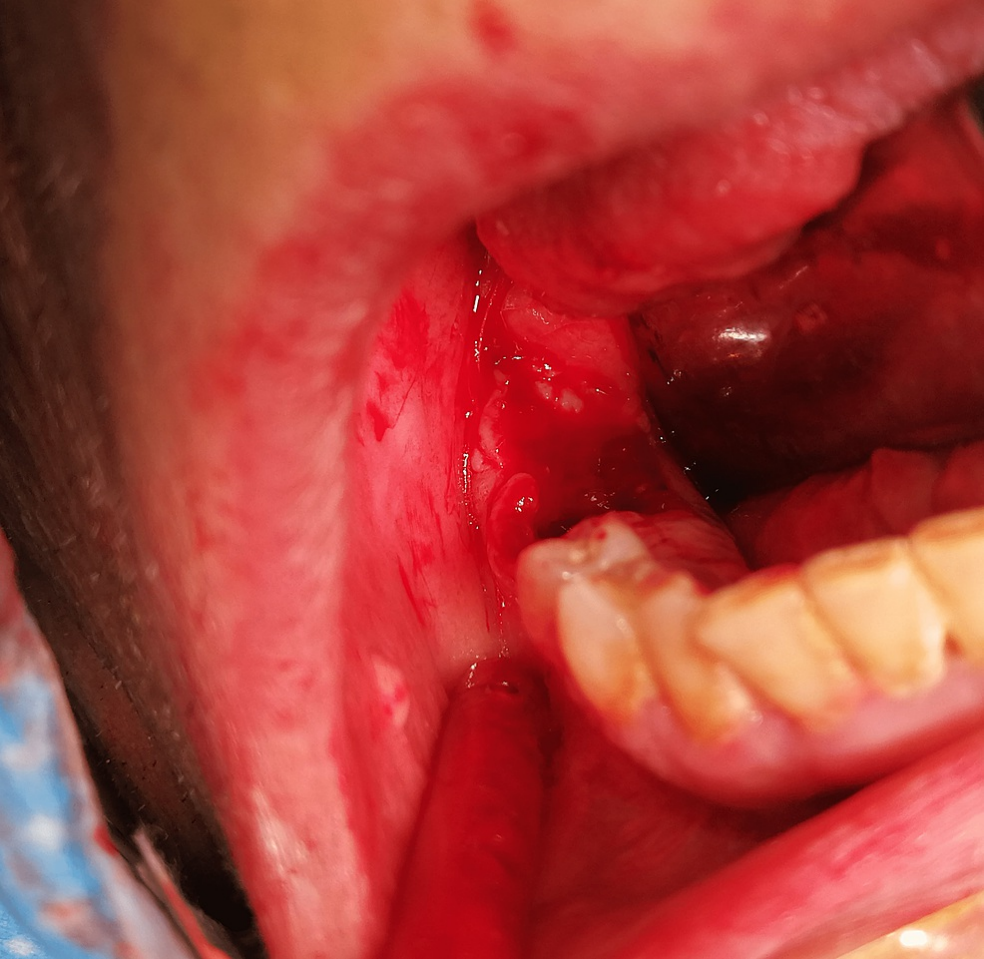
Oromaxillary defect after debridement of necrotic bone tissue.

Sequestrectomy was the treatment of choice, and the patient underwent surgical debridement under general anesthesia removing the sequestrum and extraction of teeth 47 and 48 (Figure 3). A drug holiday was decided, and bisphosphonates were discontinued one month before the procedure and restarted two months later.

**Figure 4:**
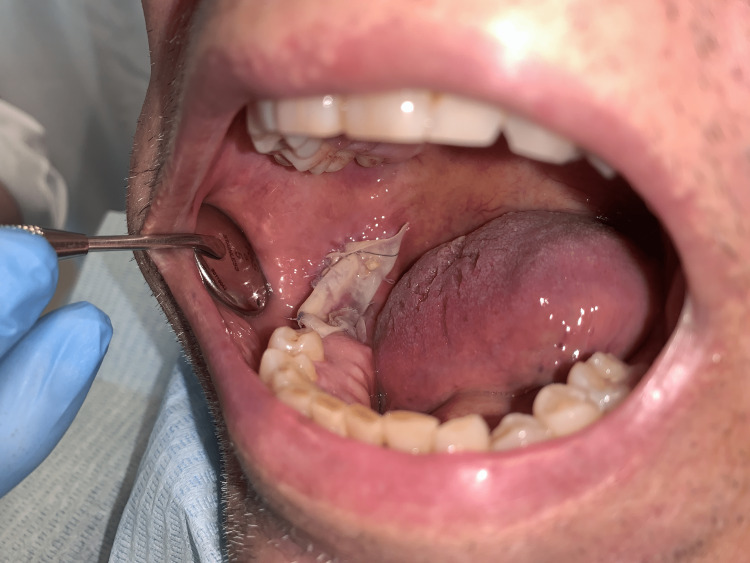
IDRT overlying the defect seven-day post-operation. IDRT = Integra® Dermal Regeneration Template

The remaining intraoral defect could not be closed directly due to a lack of soft and hard tissue following debridement and was addressed using the IDRT. A customized rectangular piece of the dual-layer matrix was placed directly onto the bone and secured with 4.0 absorbable sutures to the mucosa at the edge of the defect with attention to avoid folds. After the procedure, the patient remained on a soft diet and antibiotic prophylaxis with amoxicillin for one week. Follow-up on day seven post-surgery revealed no inflammatory signs at the surgical site (Figure 4).

**Figure 5:**
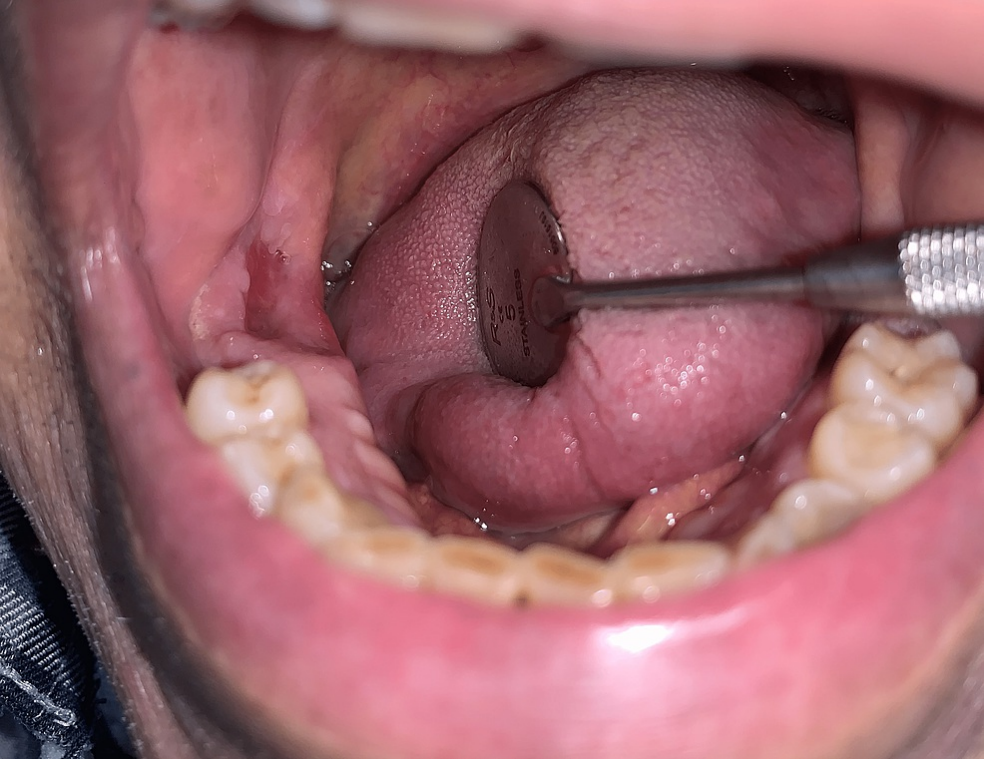
Full mucosal healing at 10 weeks after IDRT removal. IDRT = Integra® Dermal Regeneration Template

IDRT silicone layer was removed on day 21, showing early granulation over the previously exposed bone. At 10 weeks postoperatively, the oral defect had entirely healed without the need for active intervention (Figure 5).

On short-term follow-up at six months, no further MRONJ was noted.

## Discussion

In MRONJ, the application of IDRT to cover oromaxillary defects is rarely reported in the literature [6]. IDRT is a skin-replacement bilayer membrane system frequently used in burn injuries when enough autograft is unavailable or due to the patient’s medical condition. The dual-layer artificial matrix is composed of a collagen matrix of bovine origin covered with a semi-permeable silicone layer, which may allow fast wound closure and permanent dermis regeneration [4,6]. The wound-healing process begins once the IDRT membrane is applied to the surgical wound, extracellular fluids invade the matrix, and fibrin mediates the initial adhesion process. The bovine collagen matrix serves as a layout for establishing the new dermis. As wound healing progresses, cells such as lymphocytes and fibroblasts migrate to the matrix, followed by neo-angiogenesis promoted by endothelial cells. During this process, fibroblasts produce collagen, progressively replacing the bovine collagen matrix. Almost three weeks after the IDRT application, the new dermis is formed. The vascularization is enough to allow the removal of the silicon layer because the IDRT is now fully integrated, leaving a well-established autologous dermis [6].

Traditionally, the resolution of acute infection and pain in stage III MRONJ requires necrotic bone debridement or resection in addition to antimicrobial therapy [3]. Reconstruction of oromaxillary defects is possible with vascularized bone flaps, free tissue flaps, or, to fill more minor defects, a local mucosal flap, all described in the literature with varying levels of success [3,7]. In this case, the metastatic prostate cancer, the extent of necrosis, and the ongoing medical complications led to the choice of a less invasive procedure. Therefore, a decision was made to reconstruct the defect with the dual-layer membrane.

An excellent surgical technique in the IDRT application is essential to reducing pressure or tearing of the matrix [8]. Compliance with a soft diet postoperatively minimizes the risk of mechanical detachment. Both are particularly important in oral and maxillary surgery due to the forces and movements present in the stomatognathic system. In this case, this technique promoted an excellent result. The defect was covered entirely by attached gingiva, which allowed no further interventions commonly associated with increased morbidity in MRONJ patients [3].

Nevertheless, some limitations may be noted because this matrix requires a good-quality blood supply from the neighboring tissues and a thorough surgical debriding to allow the growth of the new dermis free from infection [6,9]. Other studies have reported that this technique is only successful for minor oromaxillary defects such as areas of 10-20 mm or the need to secure the IDRT in place with an acrylic splint to aid tissue development [6]. Although the defect was more significant in our case (40 mm), there was no need to apply a splint or another method due to the defect’s location and compliance with postoperative recommendations. Moreover, removing the silicone layer must be performed with caution not to remove the newly formed tissue [8]. In this case, the wound area was healed by secondary intention, but a graft can also be an option and applied over the new dermis.

## Conclusions

IDRT may represent an alternative surgical approach for patients who are not candidates for more invasive procedures. This material and technique are easy to use, less invasive, and can be performed in a one-stage procedure in a day-surgery regimen representing a suitable method to cover oromaxillary defects.
